# Endocytosis and Alzheimer’s disease

**DOI:** 10.1007/s11357-023-00923-1

**Published:** 2023-08-30

**Authors:** Łukasz Zadka, Marta Sochocka, Naomi Hachiya, Justyna Chojdak-Łukasiewicz, Piotr Dzięgiel, Egbert Piasecki, Jerzy Leszek

**Affiliations:** 1 Division of Ultrastructural Research, Wroclaw Medical University, 50-368 Wroclaw, Poland; 2 Hirszfeld Institute of Immunology and Experimental Therapy, Polish Academy of Sciences, Rudolfa Weigla 12, 53-114 Wroclaw, Poland; 3 Shonan Research Center, Central Glass Co., Ltd, Shonan Health Innovation Park 26-1, Muraoka-Higashi 2-Chome, Fujisawa, Kanagawa 251-8555 Japan; 4 Department of Neurology, Wroclaw Medical University, Borowska 213, 50-566 Wrocław, Poland; 5 Department of Histology and Embryology, Wroclaw Medical University, Chałubińskiego 6a, 50-368 Wroclaw, Poland; 6 Department of Psychiatry, Wroclaw Medical University, Wybrzeże L. Pasteura 10, 50-367 Wroclaw, Poland

**Keywords:** Alzheimer’s disease, Amyloidogenic process, Amyloid-β, Tau protein, Endocytosis, Endosome secretion

## Abstract

Alzheimer’s disease (AD) is a progressive neurodegenerative disorder and is the most common cause of dementia. The pathogenesis of AD still remains unclear, including two main hypotheses: amyloid cascade and tau hyperphosphorylation. The hallmark neuropathological changes of AD are extracellular deposits of amyloid-β (Aβ) plaques and intracellular neurofibrillary tangles (NFTs). Endocytosis plays an important role in a number of cellular processes including communication with the extracellular environment, nutrient uptake, and signaling by the cell surface receptors. Based on the results of genetic and biochemical studies, there is a link between neuronal endosomal function and AD pathology. Taking this into account, we can state that in the results of previous research, endolysosomal abnormality is an important cause of neuronal lesions in the brain. Endocytosis is a central pathway involved in the regulation of the degradation of amyloidogenic components. The results of the studies suggest that a correlation between alteration in the endocytosis process and associated protein expression progresses AD. In this article, we discuss the current knowledge about endosomal abnormalities in AD.

## Introduction

According to the Alzheimer’s Association and World Health Organization (WHO), dementia affects almost 55 million people worldwide—a number that will double by 2050. Alzheimer’s disease (AD) is the most common cause of dementia and the most prevalent in the elderly population [1]. AD is a gradual and progressive neurodegenerative disorder clinically characterized by deterioration of episodic memory and successive impairment of additional cognitive domains, with behavioral changes impacting activities of daily living. Although AD has no specific symptoms, the diagnosis is currently made by meeting ICD-10 or DSM-V criteria, which include the presence of cognitive deficits in the form of memory loss without impaired consciousness, agnosia, aphasia, apraxia, impaired executive activities, a progressive disease process, deterioration in social and occupational functioning, and changes in social behavior [2]. The debilitating course, high mortality, and potential tripling of the incidence of AD by 2060 result in an increase in global economic expenditures for the healthcare sector [3]. Although most AD cases affect patients ≥ 65 years of age [4], the disease can also occur as early as the 3rd decade of life with an early diagnosis [5]. The lack of AD-specific biomarkers makes it difficult to rapidly detect the disease, especially in the early stages of AD when patients present with initial prodromal symptoms. For a very early diagnosis of AD, we need to have affordable, ultrasensitive, and selective molecular detection methods. We have till now failed to manage it effectively, with only a handful of symptomatic therapies and questionable newer disease-modifying agents [6].

In the early 1990s, the amyloid cascade hypothesis was revealed as a primary pathway that leads to AD. Currently, the major theories related to the mechanisms involved in the pathogenesis of AD are amyloid-β (Aβ) plaques, neurofibrillary tangles (NFTs) with phosphorylated tau protein (P-tau), gliosis, and neuronal loss, accompanied by cerebrovascular amyloidosis, inflammation, and major synaptic changes. The model was based on the assumption that extracellular deposits of Aβ initiate and are a direct cause of neurotoxicity, induction of tau protein pathology—NFT formation—and handicap brain vascular system, leading to neuronal death and neurodegeneration [7]. In addition, cumulating Aβ deposits in turn activate glial cells—microglia (resident immune cells of the brain, macrophages) and astrocytes—leading to an inflammatory response in the central nervous system (CNS)—neuroinflammation [8]. More recent studies suggest that astrocyte reactivity abnormality could be placed as an early biomarker model of AD progression, which may explain the validity of anti-Aβ therapy, modifying this Aβ-astrocyte-tau pathway. There is an important emphasis on a decrease in synapse density and a corresponding decrease in brain volume due to neurodegeneration [9]. The second core pathology in AD is a deposition of hyper-phosphorylated tau protein inside neurons. AD pathogenesis involves pathogenic contributions from multiple components, signaling pathways, and alterations in the behavior of various cell types. Previous studies indicated that immune response, Aβ metabolism, cholesterol/lipid dysfunction, endocytosis, and angiogenesis are strongly associated with AD. A recent large-scale genome-wide association study (GWAS) showed a number of endocytosis-related molecules in AD, and the relationship between AD and endocytosis (e.g., in Aβ aggregation) is now of great interest to the scientific community [10].

In this work, we discuss current knowledge on the role of endocytosis in AD pathophysiology and show a potential link between endocytosis and AD.

## Types of endocytosis

**Fig. 1:**
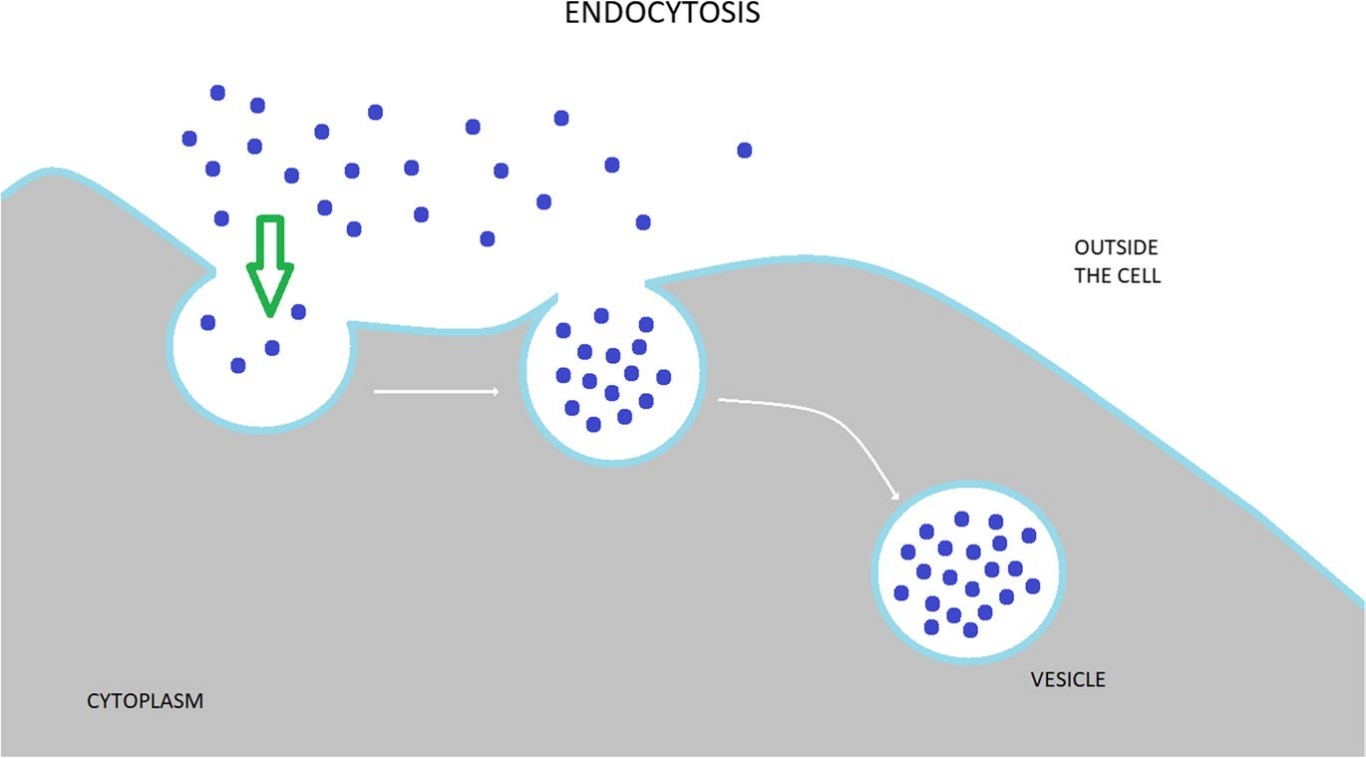
Graphical presentation representing endocytosis. In endocytosis process, various extracellular components unbound or recognized by membrane-localized receptors become transported into plasma membrane–derived vesicles. These structures may serve to recycle the material back to the plasma membrane or dispatch it to lysosomes for future degradation

Endocytosis is a fundamental cellular process in all eukaryotic cells, which regulate nearly every aspect of cellular physiology and are often impaired in pathological conditions. It is the cellular process of membrane vesicular transport between the plasma membrane (PM) and cytoplasmic membrane compartments, as well as within the intracellular membrane system (Fig. 1). Cells use endocytosis to take up different types of molecules that cannot otherwise pass through and is an essential mechanism for homeostasis and communication within and between cells. The surface proteins, lipids, and other macromolecules are enveloped within small membrane vesicles formed by the entrapment of the cell membrane and subsequently taken up into the cell. Endocytosis is a complex program that is strongly tied to signal transduction [9, 11] and serves as the cell’s primary communication infrastructure. Endocytosis also helps to generate cell polarity by rapidly redistributing cell surface molecules to active locations [12, 13]. Instead of planar diffusion across the plasma membrane, this fast and site-specific redistribution of membrane proteins is accomplished through a cycle of endocytosis and directed recycling. Although endocytosis is primarily associated with the function of individual cells, it also regulates the function of hundreds of assembled cells. This is interesting because it implies that the endocytosis event, which occurs at the cell level, may be synchronized across numerous cells and act in a coordinated manner.

Each cargo taken up by nascent endocytotic vesicles is different. The cargo transport after endocytosis will largely depend on the combination of cargo and coat proteins involved in recognizing a specific cargo [14–16]. As a result, these cargoes can be employed as markers for the respective endocytotic and post-endocytotic pathways. On the other hand, it is not rare for a specific cargo to be identified in more than one endocytosis pathway. Endocytosis is usually subdivided into phagocytosis, pinocytosis, clathrin-mediated endocytosis, and caveolin-mediated endocytosis. Pinocytosis involves the internalization of small molecules, whereas phagocytosis involves the internalization of large particles.

To maintain their characteristic polar structure, neurons require the right distribution of different receptors, the perfect arrangement of proteins and organelles in dendrites and axons, continual exocytosis/endocytosis of synaptic vesicles, and the removal of defective proteins. Because synaptic vesicles must be constantly exposed to exocytosis and endocytosis, continuous membrane trafficking is essential at synapses [17–19]. As a result, membrane transport is involved in all aspects of neuronal function, and its malfunction is linked to neurodegeneration.

### Phagocytosis

Phagocytosis is a kind of clathrin-independent endocytosis (CIE) in which membrane-derived vesicles known as phagosomes selectively detect and internalize particles with sizes greater than 500 nm. Phagocytosis is divided into four stages: target recognition, signal transduction that initiates the internalization process, phagosome formation, and phagolysosome maturation. The cytoskeleton must be involved in membrane modification during this process. Phagocytosis is a complicated process that is necessary for growth and tissue homeostasis and removing pathogens and apoptotic cells [20]. It is also the first line of defense involving innate immune cells against infections, and immune system phagocytes such as neutrophils, macrophages, monocytes, and dendritic cells use phagocytosis to consume invading organisms, pathogens, dead cells, and debris [21]. Surface receptors recognize phagocytosis targets and are divided into two types: opsonin receptors and non-opsonin receptors. Opsonin receptors detect foreign particles by binding to opsonin produced by the host. Non-opsonin receptors recognize a collection of chemicals on the surface of the phagocytosed item [21, 22]. When these receptors bind to target particles, they activate the protein tyrosine kinase Syk, which generates phosphoinositide second messengers and initiates an intracellular signaling cascade that recruits activated Rho GTPases. Small GTPases (which contain Ras, Rho, Rab, Ran, and Arf proteins) are central regulators of a wide variety of signal transduction pathways (regulate actin reorganization, influence cell polarity, microtubule dynamics, membrane transport pathways, and transcription factor activity) in all eukaryotic cells. Rho GTPases reorganize actin polymerization in phagocytic cups to form pseudopods, which seal their ends and transform into phagosomes after surrounding the target particle [23]. In the final step of phagocytosis, phagosomes continually merge and split with endocytic vesicles before fusing with lysosomes to form phagolysosomes. This process involves a continuous decrease in pH and the acquisition of digestive enzymes, which leads to target digestion and antigen retrieval for presentation on the phagocytic surface [24].

### Macropinocytosis

Pinocytosis is a type of nonspecific endocytosis in which dissolved objects are integrated into vesicles of arbitrary size. Macropinocytosis is an actine-mediated bulk type of endocytosis, a large-volume endocytic pathway that produces vesicles (macropinosomes) with diameters ranging from 200 nm to 5 µm [25]. Under the cell membrane, actin polymerization rings (circular ruffles) can form, forming a cup-like entry opening. When this cup closes, it transforms into a macropinosome. Because the cargo taken up by macropinocytosis can range from liquids to particles and can be taken up in large quantities, this pathway has piqued the interest of drug delivery researchers. Unlike phagocytosis, amiloride specifically inhibits macropinosomes by blocking the Na + /H + exchanger in the cell membrane. Pathogens have evolved sophisticated macropinocytosis mechanisms, which allow for metabolic adaptation and survival in nutrient-deficient environments [26]. Micropinocytosis is regulated by a group of small GTPases. Rab family proteins, which are small GTPases, govern cytoskeleton movement and membrane retention. A small GTPase is a molecular switch that has two states: inactive (bound to GDP) and active (attached to GTP). The activation/inactivation cycle is made up of a guanine exchange factor (GEF) that activates the GTPase by promoting GTP binding, a GTPase activating protein (GAP) that inactivates the GTPase by hydrolyzing GTP, and a guanine nucleotide that prevents GDP dissociation from the GTPase and keeps the GTPase inactive [27]. Ras GTPases activate phosphatidylinositol 3-kinase, causing the cell membrane to form a PIP3-rich membrane domain. This membrane domain functions as a docking site for Rho GTPases and promotes membrane ruffling through actin remodeling. The maturation process is mediated by small Rab GTPases and phosphoinositides once the macropinosome is closed at the plasma membrane. The macropinosome fuses with the early endosome initially. Rab5 and Rab34 facilitate fusion, and Rab5 changes to Rab7 during macropinosome maturation, encouraging fusion to the late endosome/lysosome compartment [28–30].

## The dysregulation of endocytosis in AD

Evidence from both genetic and biochemical studies supports the involvement of endosomal abnormalities in the pathogenesis of AD. Many endosomal pathways play a role in the amyloidogenesis process. The results are not always unambiguous. In addition, it remains essential to answer the question of whether the changes are the result of the cause of developing AD.

### Caveolin-mediated endocytosis

**Fig. 2:**
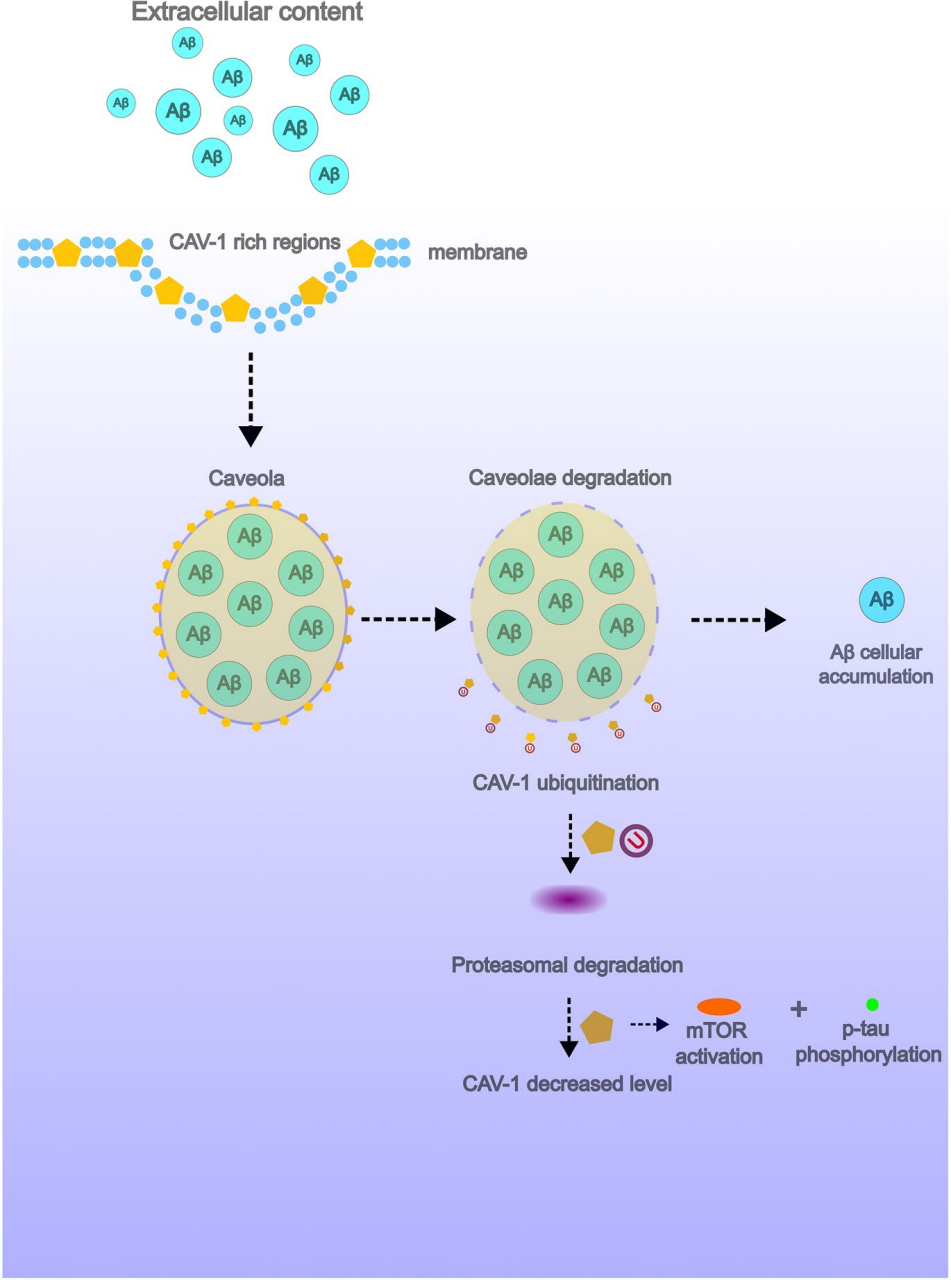
Hypothesized model of endocytosis pathway in Alzheimer’s disease (AD). In AD, endocytosis resulted in forming increased deposition of Aβ peptides by omitting lysosomal digestion of processed endosomes. Increased activation of mTOR signaling leads to inhibition of CAV-1 expression and hyperphosphorylation of the tau protein degradation of CAV-1-rich caveolae resulting in decreased level of this marker in AD-affected brain tissue

The caveolar pathway involves caveolae, which are bulb-shaped, 50–60-nm plasma membrane invaginations. Caveolae as cholesterol-rich microinvaginations in the cell membrane are associated with the production of Aβ peptides and may play a role in the pathogenesis of AD [31]. However, the results obtained are inconsistent. A positive immunoreactivity to caveolin-1 (CAV-1) is commonly observed in these endocytic structures. In aging brain tissues, CAV-1 was overexpressed, the distribution of which was particularly pronounced in the neurons of the II-VI cortical layers, mainly in pyramidal cells and in the pyramidal cell layer of the CA1 region of the hippocampus, and its subcellular localization at the ultrastructural level was observed in the 50–200-nm-sized vesicle-like caveolae structures. Moreover, the increased level of CAV-1 had a negative effect on the expression of alpha-secretase-released APP ectodomain (sαAPP), probably by blocking the phosphorylation of protein kinase C (PKC) [32]. Contrary to these results, in the post-mortem brain tissue from AD cases, CAV-1 expression was not significantly higher than in control tissues; however, the abundant accumulation of Aβ in the walls of cerebral vessels was associated with loss of CAV-1 and caveolin-2 (CAV-2) expression [56]. There were also changes in the distribution and phenotype of these structures associated with the AD pathological signs. In post-mortem studies of the human brain tissue, accumulation of the voltage-dependent anion channel (VDAC) was observed in caveolae, the distribution of which was particularly pronounced in vanishing neurites of senile plaques, and estrogen receptor alpha (ERα) expression was noted mainly in astrocytes surrounding Aβ plaques [33]. Caveolae may play an important role in the enhanced capture of extracellular Aβ peptides. The inducing of the caveolae-mediated endocytosis allows to bypass the lysosomal degradation of the transferred load, which increases the efficiency of the endocytic cargo transfer mechanism [34]. Changes in the expression level of caveolae markers and AD-related pathological molecules are also influenced by certain risk factors for this dementia. High glucose levels induced increased activation of mTOR signaling, which led to inhibition of CAV-1 expression and hyperphosphorylation of the tau protein [35]. The hypothetical importance of caveolae in the pathogenesis of AD has been demonstrated (Fig. 2).

### Clathrin-mediated endocytosis (CME)

**Fig. 3:**
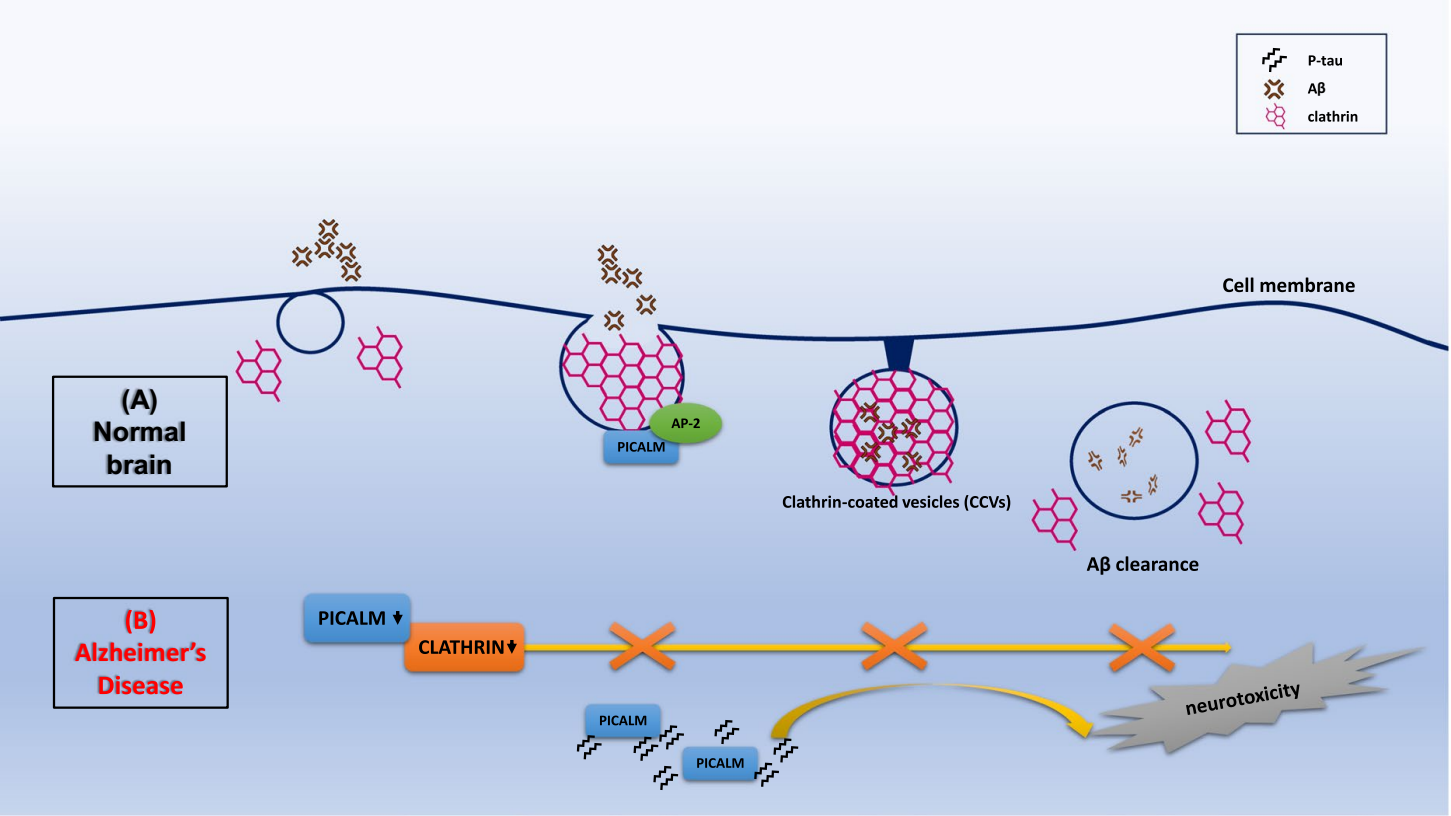
Regulation of clathrin-mediated endocytosis (CME) by PICALM. (A) In a healthy brain, PICALM interacts with adaptor protein (AP) and clathrin at the plasma membrane. PICALM participates in CME and facilitates the formation of clathrin-coated vesicles (CCVs). This provides Aβ clearance. (B) PICALM and clathrin depletion in AD brain limited or stopped Aβ clearance. This increases the distribution of Aβ, intensifying neurotoxicity to neurons. In AD, abnormally cleaved PICALM was also associated with neurofibrillary tangles, co-localizing with conformationally abnormal and hyper-phosphorylated tau (P-tau) which contributes to endocytic dysfunction in AD

Clathrin-mediated endocytosis (CME) is the major endocytic pathway for the internalization of numerous cargos. In a mouse AD model, the cortical overexpression was confirmed for clathrin-mediated endocytic proteins such as clathrin, dynamin II, and some phosphatidylinositol-binding clathrin assembly protein (PICAM) variants [36]. PICALM belongs to endocytic-related proteins with important regulatory action in the process of endocytosis. Silencing the expression of PICALM isoforms decreased the levels of intracellular amyloid precursor protein (APP), intracellular β-C-terminal fragment (β-CTF), and soluble peptide APPβ (sAPPβ) in H4 cells but had no effect on Aβ_40_. PICALM and clathrin depletion had a negative effect on the endocytosis process and decreased the expression of beta-secretase (BACE1) at the mRNA level; however, only silencing PICALM expression inhibited BACE1 at the protein level and negatively influenced the cytoplasmic level of clathrin [37]. PICALM as the key clathrin adapter protein was an important mediator of endocytosis for the CME pathway of which the expression at the protein level was decreased in AD brain homogenates, but its immunoreactivity showed a clear cellular differentiation between AD cases and control tissues, showing a clearly higher level of expression in microglia and in neurons in late-onset AD. Moreover, the expression of PICALM in AD was characterized by co-localization with NFT and P-tau protein, which was especially crucial for vanishing neurites and the vicinity of Aβ plaques [38]. PICALM was responsible for endocytosis in the CME pathway associated with the low-density lipoprotein receptor–related protein-1 (LRP1), which regulated Aβ trafficking to Rab5 and Rab11 ensuring proper amyloid clearance. In AD, the reduction of PICALM expression in endothelial cells limited transcytosis and Aβ clearance [39]. PICALM protein is a key molecule in the regulation of the CME mechanism, which in turn increases the distribution of clathrins and adaptor protein 2 (AP-2) in the regions of cell membranes, which facilitates the formation of clathrin-coated vesicles (CCVs). In the AD brain, PICALM was expressed mainly in endothelial cells, which may affect the removal of Aβ through the brain vascular walls [40] (Fig. 3).

AP-2 as one of the clathrin’s adaptor proteins may have an influence on the phenomenon of APP endocytosis in the AD brain [41]. Overexpression of AP-2 occurring under the influence of a high-fat diet increased the formation of BACE1/AP-2/clathrin complexes on the surface of cell membranes and induced the redistribution of the resulting complex into the cytoplasm, which promoted APP cleavage [42] and may increase the distribution of Aβ, intensifying neurotoxicity to nervous tissue. The phenomenon of Aβ transcytosis via the brain endothelial cells was caused, among others, by the ABC transporter P-glycoprotein (ABCB1/P-gp) and LRP1 which, in a mechanism dependent on PICALM, regulated the outflow of Aβ through the blood–brain barrier (BBB) [43]. CME via Ca^2+^ signaling may induce axonal damage resulting from the increased distribution of Aβ peptides, while inhibition of CME in both the in vivo and cellular culture models inhibited axon loss and improved cognitive functioning in the mouse AD model [44]. The amyloidogenic process of APP, which produces Aβ peptide, occurs in an intracellular compartment requiring endocytic trafficking [45–47]. APP is processed by BACE1 in Rab5-positive early endosomes under physiological conditions, resulting in a β-CTF [48, 49]. This β-CTF is converted to an Aβ peptide at late endosomes and the Golgi [50]. This emphasizes the relevance of GTPases and membrane trafficking in the etiology of AD. Furthermore, numerous genes associated with endocytic trafficking are linked to the likelihood of acquiring AD [51–56].

During cellular senescence in neurons, APP endocytosis was potentiated and Aβ secretion was increased, due to the higher distribution of F actin and clathrin. Other changes observed at the cellular level included the increased formation of early endosomes, APP co-localization to nascent endosomes, and degeneration of synaptic connections associated with the endocytic generation of Aβ progressing with age [57]. Endocytosis in the CME pathway mediated by γ-secretase internalized by clathrin assembly lymphoid myeloid leukemia protein (CALM) through endosome formation regulated Aβ_41_ production is trafficking by retrograde transport via the endosome-to-trans-Golgi network (TGN) pathway [58]. The loss of CALM disrupted γ-secretase-mediated APP processing leading to reduced release of Aβ_42_ by HeLa cells [59]. Another possibility related to the increased Aβ distribution in the AD brain is autophagy. The expression of proteins that regulate autophagy was changed during cellular senescence. LC-3-associated endocytosis is an essential process for the processing of Aβ receptors, including TREM2, which in effect ensured protection against the formation of Aβ plaques and the formation of cognitive deficits in an AD animal model [60]. One of the potential causes of the cognitive deficit observed in AD patients was the CME pathway–related loss of AMPA receptors (AMPARs) in the postsynaptic regions, induced by the intensification of endocytosis due to Aβ plaques [61]. AMPA-type glutamate receptors (AMPARs) are a group of receptor protein complexes that regulate neuroplasticity and synaptic transmission related to the learning and memory processes. The endocytic adapter CALM and AP180 N-terminal homology (ANTH) domain–containing proteins HIP1 and HIP1R regulated Ca^2+^-permeable GluA1 AMPAR homomer endocytosis by affecting the membrane expression of these receptors in the postsynaptic region of neurons through membrane remodeling by a clathrin mechanism independent of clathrin [62].

### Microglia acting in the AD-affected brain via phagocytosis and clathrin-independent pathway

**Fig. 4:**
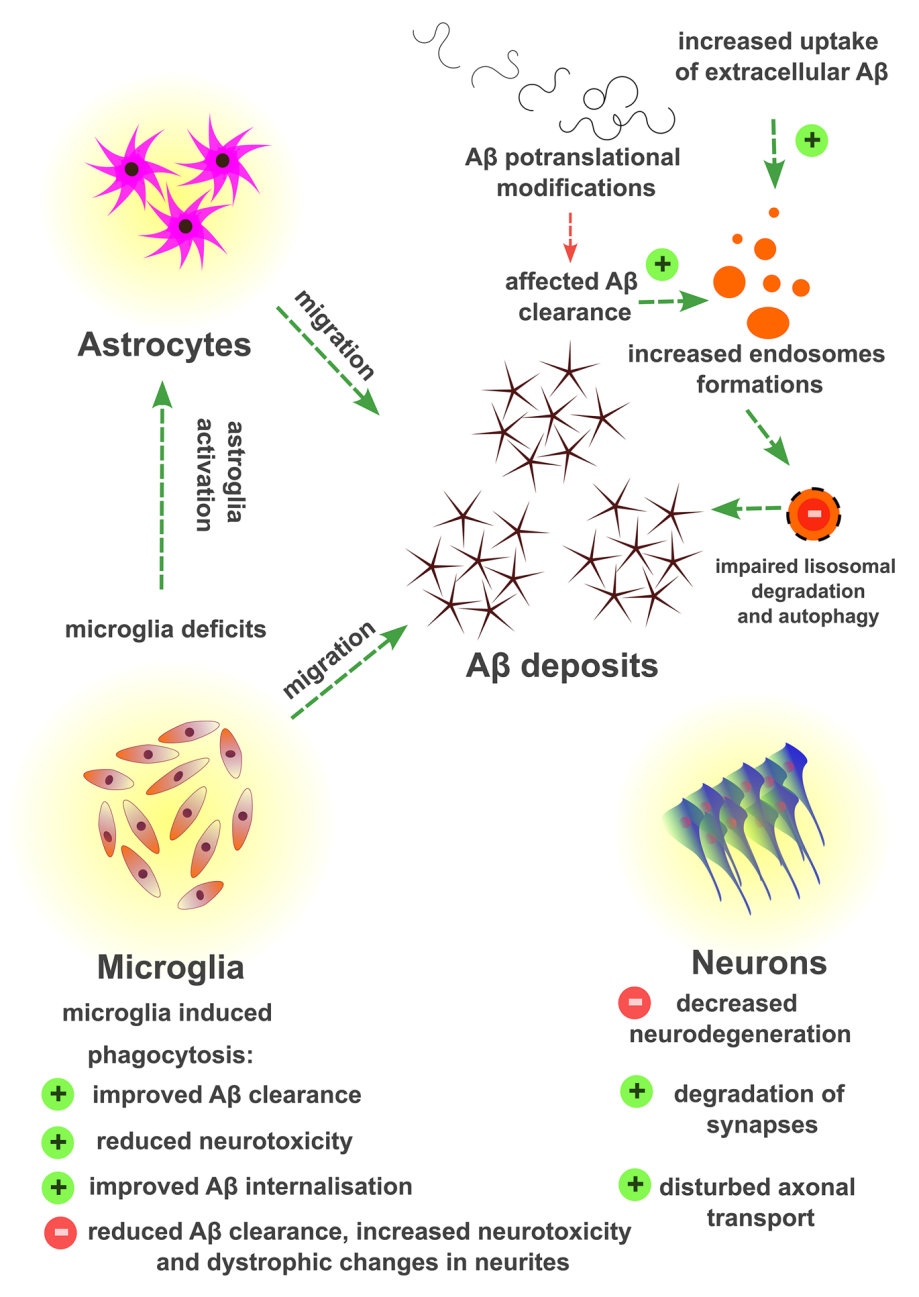
Graphical presentation representing the theoretical basis of Aβ accumulation in the brain tissue. Aβ deposits in the brain cause the migration of astrocytes and microglial cells into the region of plaque formation. Biological task of these cells is to phagocytose the formed lesions. Heterogeneity in proteolytic cleavages during Aβ generation and the relevant post-translational modification processes of Aβ may translate into the rate of amyloid aggregate formation, their neurotoxicity, and the effect of plaques against phagocytic cells. The symbols of the stop sign and the plus sign refer to the intensity of a given process or to the effect of increased or decreased endocytosis in relation to selected types of cells making up the nervous tissue

Phagocytosis is one of the subtypes of endocytosis in which cargo assimilation can occur regarding the receptor-mediated or non-receptor-mediated pathway [63]. Aβ deposits in the brain activate the local immune response, which leads to the migration of astrocytes and microglial cells into the region of plaque formation, whose biological task is to phagocytose the formed lesions. Nevertheless, amyloid peptides that accumulate in the AD brain may impair the phagocytic capacity of astroglial cells to remove the produced Aβ oligomers [64]. Phagocytosis of Aβ plaques by microglia changes over time the gene expression profiles of late-onset AD and modulates the activity of these cells toward increased phagocytosis of synaptic components located in the vicinity of the plaques [65]. The generation of Aβ peptides was characterized by high heterogeneity in proteolytic cleavages and in the relevant post-translational modification processes, which may translate into the rate of amyloid aggregate formation, their neurotoxicity, and the effect of plaques against phagocytic cells (Fig. 4).

Post-translational modifications of Aβ peptides related to their phosphorylation seem to hinder phagocytosis of amyloid deposits by microglial cells in a TREM2-dependent mechanism [66]. TREM2 as a membrane-associated receptor is located not only in microglia cells, but also in macrophages and dendritic cells, while its influence on phagocytosis seems to depend on the level of its expression and the time of exposure to microglia. Decreased TREM2 expression in a mouse model led to the deposition of Aβ in late-stage AD, a decline in plaque-associated microglia, and caused the downstream of tau phosphorylation [67]. The phenotype of microglia may be altered in the pathogenesis of AD, which also plays a role in the activity of these cells. The dietary antioxidants such as cyani-din-3-O-glucoside (C3G) can change the direction of microglia polarization from the pro-inflammatory M1 phenotype to the M2 phenotype by overexpression and activation of PPARγ and subsequent phagocytic removal of Aβ_42_ peptides associated with TREM2 overexpression [68]. The phagocytic activity of microglia toward tau was also dependent on some dietary components, such as α-linolenic acid (ALA), which intensified the phagocytosis process. Microglia also showed the potential for phagocytosis of the extracellular tau protein; however, the rate of this phenomenon depended on the ability of these cells to migrate and induce the repolarization of the microtubule-organizing center [69]. Microglial activity in a mouse animal model of AD was focused in the vicinity of Aβ plaques and was associated with increased expression of phagocytic regulating factors such as TREM2 and CTSD, which improved the Aβ clearance and thus reduced neurotoxicity to neural cells [70]. Ren and colleagues showed that in a mouse animal model, the deficit of microglial cells led to a decrease in Aβ phagocytosis, increased distribution of Aβ peptides, increased activity of glial cells, and intensified dystrophic changes in neurites, which was related to the acceleration of cognitive impairment [71]. Moreover, microglial cells in AD clean the parenchyma of brain tissue by phagocytic removal of fibrillary Aβ and elimination of soluble Aβ (sAβ) peptides by internalizing these molecules from the extracellular space through macropinocytosis [72].

### Phosphatidylinositol-4,5-bisphosphate (PIP_2_) and AD endocytosis

Aβ deposits may impair synaptic transmission leading to cognitive deficits in AD by hydrolysis of PIP_2_ [73]. Familial AD (FAD)-associated presenilin mutations affected PIP_2_ metabolism. The cellular level of PIP_2_ had a positive effect on the functional potential of the transient receptor potential melastatin 7 (TRPM7)-associated Mg^2+^-inhibited cation (MIC) channels and is inversely correlated with the level of Aβ_42_ peptides [74]. Mutations in the *PLCG2* gene were related to PIP_2_ depletion and decreased level of this marker, which in vivo showed a relationship with reduced phagocytosis and increased endocytosis expressed by acceleration of the clearance of Aβ_1–42_ oligomers [75]. Changes in PIP_2_ expression may also depend on the exogenous dietary factors. In an animal model, cerebral overexpression of phospholipase C (PLβC1) induced by high dietary cholesterol levels led to decreased PIP_2_ expression [76]. High cholesterol levels affect the excitability of neural cells through a potential affinity for the PIP_2_ transmembrane domain that stabilizes its interaction with G protein–activated inward rectifier potassium channel 2 (GIRK2) [77]. PIP_2_ through its effect on capillary endothelial Kir2.1 channels positively influenced the cerebral blood flow, which often remains impaired in AD brain [78].

### Regulation of endosome secretion and AD pathogenesis

The mechanism of the formation of Aβ aggregates in AD was dependent on the endocytosis of the APP through YTSI, the motif that promotes early endosome formation, which positively regulated this process by binding to APP tail 1a (PAT1a) and subsequent overexpression of RME-6, which was an activator of Rab5 [79]. GTPase Rab5-positive APP-carrying endocytic vesicles have been described in a rat brain model. In vesicular organelles, the highest frequency of Rab5-positive structures was found in the small synaptic vesicles (SSVs), large bilamellar vesicles (LLVs), and the lowest in multivesicular bodies (MVBs) [80]. Changes in the expression level of proteins of the Rab GTPases family were noted in the AD brain tissues. Examination of post-mortem human brain tissue homogenates revealed elevated levels of Rab5 and Rab7 proteins in the basal forebrain, frontal cortex, and hippocampus regions in AD patients compared to normal brain tissues [81]. The number and size of the formed Rab5-positive endosomes depended on the exogenous expression of APP (V642I) and the APP-binding protein APP–BP1 and increased the level of Rab5 and early endosomal antigen 1 (EEA1) proteins, which also were elevated and carried in early endosomes [82]. Moreover, the Rab5 inhibition increased the release of Aβ_40_ and Aβ_42_ peptides in N2a cell cultures [83]. Other endosome-related proteins may also mediate the pathogenesis of AD. Overexpression of Ras and Rab interactor 3 (RIN3) led to the accumulation of APP carboxyl-terminal fragments (CTFs) and increased tau protein phosphorylation, which in the AD model led to enlarged early endosome secretion [84]. The increased level of β-CTF induced the recruitment of a protein adapter containing pleckstrin homology domain, phosphotyrosine-binding domain, and leucine zipper motif (APPL1) to Rab5 endosomes, which resulted in intensification of the endocytosis phenomenon, including impaired axonal transport and endosome edema [85]. The retromer complex plays a key role in regulating endosome sorting processes with respect to the endosome-to-Golgi retrieval pathway and the endosome-to-cell-surface recycling pathway, and increased Rab7a activity dependent on TBC1D5 protein inhibition promoted recruitment of retromer cargo selective complex (CSC) to endosomes [86].

### Sorting nexins (SNXs) and AD-associated endocytosis

Sorting nexins (SNXs), which function as adaptor proteins, sort a variety of protein cargoes via the endolysosomal system to promote intracellular protein transport and signaling [87–92]. SNXs are made up of “membrane-bound” sorting dimers (SNX1, SNX2, SNX5, SNX6) and vacuolar protein sorting trimers with a membrane curvature detection domain (BAR domain) (Vps26, Vps29, Vps35) [21–23]. In a yeast-2 hybrid screen, SNX1 was found to interact with the epidermal growth factor receptor (EGFR), making it the first SNX found in mammals [93, 94]. SNX belongs to a protein family and features a conserved phosphoinositol-binding domain (PX domain) [95]. When the PX domain connects to PIP, SNX can bind to PIP-rich regions of the endocytosis network [96]. Abnormalities in SNX family proteins are associated with pathologies of the central nervous system. In particular, aberrant expression of SNX and mutations in autosomal recessive genes cause cerebellar ataxia, intellectual disability syndrome, AD, PD, and Down syndrome (DS) [91, 97–99]. Interestingly, in the DS mouse model, restoring hippocampal levels of SNX27 restores synaptic and cognitive deficits [100, 101]. SNX dysfunction has also been seen in epilepsy and schizophrenia [102]. Furthermore, changing SNX protein expression is linked to endocytosis, the basis of neural function and synaptic plasticity, and modulates complex behaviors such as learning and memory [103]. As a result, an improved understanding of SNX’s role in neuropsychiatric and neurodegenerative illnesses necessitates a reconsideration of how SNX maintains and inhibits normal brain function. SNX1 shares similarities with Vps5, a recognized yeast retromer complex component [104]. Retromer complexes are “hetero-pentameric” complexes that enable cargo transit and recovery from the endolysosomal system to the plasma membrane and trans-Golgi network (TGN), implying that SNX participates in cargo recovery from the degradation route [105–109].

SNXs may regulate distinct signaling pathways with divergent effects on the pathogenesis of AD. Markers modulating endocytosis may be related to APP shedding related to the pathogenesis of AD. SNX33 is one of the endocytic proteins that binds to dynamin through the SH3 domain. This resulted in the inhibition of endocytosis expressed by an increase in α-secretase cleavage and inhibition of both transferrin uptake and APP endocytosis [110]. SNX3 overexpression inhibited the APP uptake in the cell culture model, which led to an increase in the level of the precursor on the cell membrane and led to an increased cellular distribution of the full-length APP. SNX3 overexpression decreased the production of Aβ_40_, Aβ_42_, and sAPPβ [111]. Sorting nexin-4 (SNX4) overexpression led to increased levels of BACE1 and Aβ, which were presumed to be associated with an increase in BACE1 half-life time and with an enhanced Aβ release. The expression of SNX4 was impaired in the AD brain, emphasizing the dynamic distribution of the protein depending on the duration of the disease. In the early stages, the increased expression of this nexin was noted, and in the late stage of AD, its level significantly decreased [111]. SNX4 expression was common in nervous tissue, and its neural localization at early and recycling endosomes as well as in the synaptic regions was related to the regulation of neurotransmitter production and the release and transport of synaptic vesicles [112]. The biological importance of SNX4 complexes was related to the recognition and sorting of the charge on endosomal regions of cell membranes and was one of the pathways responsible for the proper functioning of the endosome [36]. Knockdown of sorting nexin 6 (SNX6) increased the levels of BACE1, sAPPβ, and CTFAβ in SK-N-SH and HEK293 cells [113]. Sorting nexin 12 (SNX12) was also expressed in nervous tissue, its localization at the ultrastructural level of the cell concerned mainly early endosomes, and overexpression of this nexin significantly decreased the levels of Aβ, soluble APPβ, and APPβ-carboxyl-terminal fragments. In the AD brain, a decreased level of SNX12 was noted, the interaction of which with BACE1 affected Aβ clearance [114].

## Conclusions and future perspectives

The causes of AD remain unknown. The etiology is based on two main hypotheses: amyloid plaques and neurofibrillary tangles. Additionally, several risk factors such as increasing age, genetic factors, head injuries, vascular diseases, infections, and environmental factors play a role in the pathogenesis of the disease. Besides the well-established AD pathogenesis processes, there is an increasing role of endocytosis in AD pathophysiology. Endocytosis is a pathway involved in the production, trafficking, and clearance of Aβ. Endolysosomal abnormalities occur within neurons in AD, and they are linked to both Aβ and tau pathologies. Upregulation in the endocytosis process with other factors could predispose to develop AD by increasing the internalization of APP and thus its subsequent metabolism to generate Aβ deposits. Hyperphosphorylation of tau protein may accelerate endocytic dysregulation. Microglia cells are activated by Aβ and secrete neurotoxic molecules. In contrast, microglia use phagocytosis to reduce neurotoxicity and improve Aβ internalization and the clearance of Aβ.

Aging and genetic risk factors for Alzheimer’s also influence endocytosis. Experimental data suggest that during cell aging, the endocytosis of APP increases [115]. Revving up of APP processing leads to Aβ over-production. If APP endocytosis is increased with neuronal aging, it should be emphasized to develop an inhibitor that does not interfere with synaptic vesicle endocytosis. Potential drugs should prevent amyloid accumulation and synaptic decline with aging. Thus, regulating APP trafficking may be a therapeutic strategy to prevent AD [115]. Understanding endocytosis processes (neuron-specific functions of endocytosis and also autophagy) and their regulatory systems can help to recognize the pathophysiology of AD. Focusing on how exactly genetic alterations in specific endocytosis protein genes interfere with neuronal survival should also be investigated. Therefore, as we presented, the endocytosis may be a target for therapeutic interventions aimed at stopping or slowing the pathogenesis of AD.
